# Super-mini PCNL (SMP) with suction versus standard PCNL for the management of renal calculi of 1.5 cm–3 cm: a randomized controlled study from a university teaching hospital

**DOI:** 10.1007/s00345-024-04954-x

**Published:** 2024-04-24

**Authors:** Sanket Kankaria, Kasi Viswanath Gali, Arun Chawla, Sunil Pillai Bhaskara, Padmaraj Hegde, Bhaskar Somani, Jean de la Rosette, Pilar Laguna

**Affiliations:** 1 Department of Urology and Renal Transplant, Kasturba Medical College, Manipal, Manipal Academy of Higher Education, Manipal, 576104 Karnataka India; 2 Department of Urology, University Hospital Southampton, Southampton, UK; 3 Department of Urology, Istanbul Medipol University, Istanbul, Turkey

**Keywords:** Super-mini, Standard, Miniaturized, Suction, PCNL, Stones, Renal, Stone clearance, Clavien–Dindo complications

## Abstract

**Purpose:**

To assess the safety and efficacy of super-mini PCNL (SMP, 14 Fr) when compared to standard PCNL (sPCNL, 24–30 Fr) in the management of renal calculi of size ranging from 1.5 to 3 cm.

**Methods:**

From February 2021 to January 2022, a total of 100 patients were randomized to either SMP group or sPCNL group in a 1:1 ratio (50 in each group) using computer-generated simple randomization. Demographic data, stone characteristics, operative times, perioperative complications, blood transfusions, postoperative drop in haemoglobin, postoperative pain, duration of hospital stay and stone-free rates were compared between the two groups.

**Results:**

Mean stone volume (2.41 cm^2^ vs 2.61 cm^2^) and stone-free rates (98% vs 94%, *p* = 0.14) were similar in both the SMP and sPCNL groups, respectively. The SMP group had significantly longer mean operative times (51.62 ± 10.17 min vs 35.6 ± 6.8 min, *p* = 0.03). Intraoperative calyceal injury (1/50 vs 7/50, *p* = 0.42) and mean postoperative drop in haemoglobin (0.8 ± 0.7 g/dl vs 1.2 ± 0.81, *p* = 0.21) were lower in the SMP group, but not statistically significant. SMP group showed significantly lower mean postoperative pain VAS scores (5.4 ± 0.7 vs 5.9 ± 0.9, *p* = 0.03) and mean duration of hospital stay (28.38 ± 3.6 h vs 39.84 ± 3.7 h, *p* = 0.0001). Complications up to Clavien grade 2 were comparable, with grade ≥ 3 complications higher in the standard group, but not statistically significant.

**Conclusion:**

Super-mini PCNL is equally effective as standard PCNL in treating renal calculi up to 3 cm, with significantly reduced postoperative pain and duration of hospital stay and lower risk of Clavien grade ≥ 3 complications, although with higher operative times.

## Introduction

Recent advancements in miniaturization of percutaneous lithotomy (PCNL) have emerged in the past few years with the aim of reducing haemorrhagic complications associated with standard PCNL [1, 2]. The ultimate goal is to achieve complete stone clearance with minimal morbidity.

Super-mini PCNL (SMP) (14 Fr) was introduced with the unique feature of active suction, differentiating it from the other miniaturized PCNL techniques, which allows for efficient stone debris extraction, providing excellent visualization while maintaining low intrarenal pressure. Following its introduction, the SMP system was found to be safe and effective for treating stones smaller than 2.5 cm [3].

This study was conducted with the aim of assessing the safety and efficacy of SMP when compared to standard PCNL (sPCNL, 24–30 Fr) in the management of renal calculi of size ranging from 1.5 to 3 cm. Primary outcome assessed was the SFR and secondary outcomes included operative time, haemoglobin drop, intraoperative and postoperative complications, postoperative pain and duration of hospital stay.

## Materials and methods

A randomized control study was carried out at a university teaching hospital, enrolling patients with renal calculi in the range of 1.5–3.0 cm based on predefined inclusion and exclusion criteria. Ethical committee approval and written informed consent from participants were obtained. The study is registered with the Clinical Trials Registry—India (CTRI/2021/02/030949). A total of 127 patients were screened, of which 100 patients were recruited and prospectively randomized into either group 1 (super-mini PCNL) or group 2 (standard PCNL) in a ratio of 1:1 using computer-generated simple randomization method. Neither the patients nor study authors were blinded to the procedure.

Inclusion criteria involved a single renal calculus measuring 1.5 cm–3 cm, irrespective of location in the kidney. Exclusion criteria involved age < 18 years, uncorrected coagulopathy, anomalous kidneys, prior percutaneous renal procedures and active urinary tract infections.

**Fig. 1:**
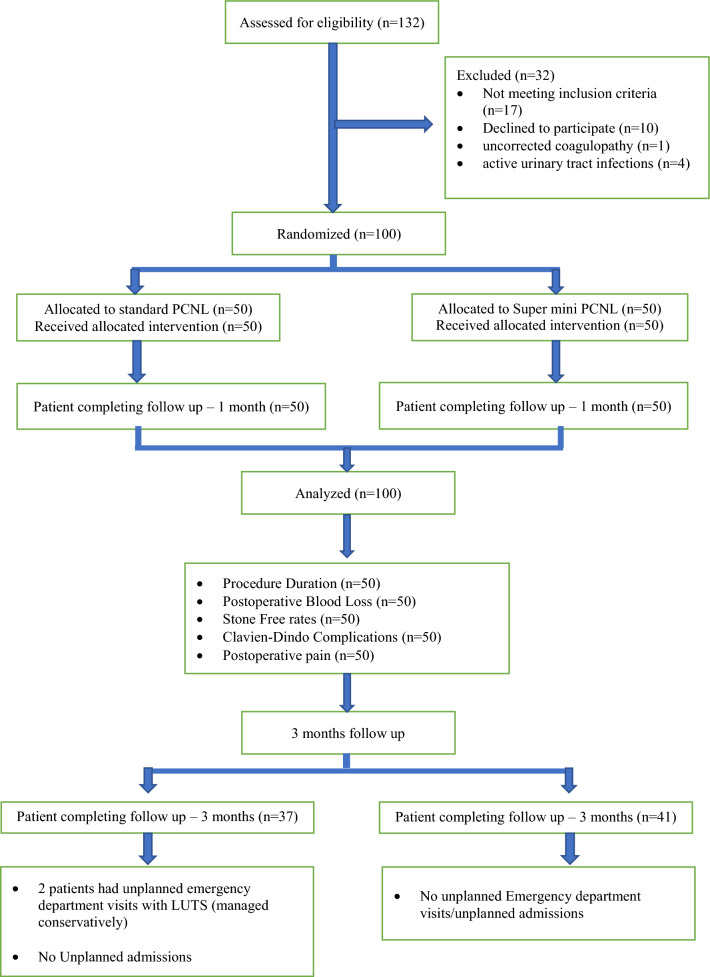
CONSORT diagram and patient disposition

Patients were evaluated upon admission for surgery under anaesthesia, with detailed history and clinical assessment. Preoperative non-contrast CT imaging and IV antibiotic administration were standard procedures. All patients underwent PCNL in the prone position under general anaesthesia. Fluoroscopy-guided calyceal puncture was performed to establish access (Fig. 1).

*Standard PCNL* involved tract dilation with sequential coaxial Alken metal dilators up to 24–30 F, securing an Amplatz sheath. Stone was fragmented using pneumatic lithotripsy/holmium:YAG laser/thulium fibre laser, and retrieval performed using graspers.

*Super-mini PCNL* utilized a single-step dilatation over the guidewire with a metal dilator, introducing the SMP sheath (a first-generation SMP from R.K.Medical devices, Mumbai, India) over the dilator. Stone fragmentation/dusting was achieved using holmium:YAG laser/thulium fibre laser. A 3 Fr grasper facilitated the extraction of residual stone fragments. Suction in SMP was intermittent, controlled by the surgeon’s finger.

### Laser settings comprised: holmium laser for fragmentation (1.2 J × 8 Hz) and dusting (0.6 J × 18 Hz); Thulium fibre laser for fragmentation (2 J × 7.5 Hz) and dusting (0.10 J × 120 Hz).

Following fragment retrieval in both procedures, nephroscopic and fluoroscopic clearance was confirmed. A double J stent was then placed as per surgeon’s preference. A single experienced surgeon performed both procedures. Data regarding puncture, access, operative time (time taken from puncture to end of the procedure), intraoperative findings, postoperative haemoglobin drop, hospital stay duration, stent/nephrostomy placement, visual analogue scale (VAS) pain scores, analgesia requirement, postoperative complications (per modified Clavien–Dindo classification) and follow-up assessments at 1 and 3 months were systematically collected. The stone-free rate, indicating the absence of fragments or clinically insignificant fragments measuring < 2 mm, was determined through a combination of X-ray of kidney–ureter–bladder (KUB) and ultrasound scan at the 1-month follow-up.

Statistical analysis was done using SPSS version 16.0. Statistical analyses utilized a t test for normally distributed continuous variables and a Mann–Whitney *U* test for those without a normal distribution. Categorical variables were assessed using either the Chi-squared test or Fisher’s exact test. A *p* value less than 0.05 was considered as statistically significant.

## Results

The patient demographics and stone characteristics were comparable in both groups (Table 1). The mean BMI (27.5 kg/m^2^ vs 27.72 kg/m^2^, *p* = 0.44) and mean stone size (2.3 ± 0.7 cm vs 2.6 ± 0.5 cm, *p* = 0.56) were similar in both the groups. All patients had a single access tract. The tract sizes in the standard PCNL arm were 28 Fr (*n* = 22), 30 Fr (*n* = 27) and 26 Fr (*n* = 1). The tract size in the SMP arm was 14 Fr (*n* = 50).

**Table 1:** Patient demographics and stone characteristics in the two study groups

Characteristics	Standard PCNL	SMP	
Mean age	48 years	43.8 years	
Male:female ratio	2.3: 1	6: 1	
BMI	27.5 kg/m^2^	27.72 kg/m^2^	
Comorbidities			*P* value
DM	13	6	0.07
HTN	12	8	0.31
IHD	3	1	0.30
Stone characteristics			
Mean stone size	2.6 cm	2.3 cm	
Mean stone volume	2.61 cm^2^	2.41 cm^2^	
Mean stone density	1107 HU	1023 HU	
Laterality—right:left	32: 18	30: 20	
Site of stone—pelvis	32	28	
Site of stone—lower calyx	12	17	
Site of stone—middle calyx	5	3	
Site of stone—upper calyx	1	2	
Supracostal puncture	8	12	
Infracostal puncture	42	38	
Upper calyceal access	10	14	
Middle calyceal access	9	5	
Lower calyceal access	31	31	

*BMI* body mass index, *DM* diabetes mellitus, *HTN* hypertension, *IHD* ischaemic heart disease

Comparative statistics of standard PCNL and SMP are stated in Table 2. Mean operative time was significantly higher in the SMP group (51.62 ± 10.17 min vs 35.6 ± 6.8 min, *p* = 0.03). Calyceal injuries were noted in seven patients in the standard group and one patient in the SMP group. Of the seven patients in the standard group, five patients had minor mucosal injury with absence of bleeding, one patient had major mucosal laceration with the presence of bleeding and one patient had calyceal injury with peri-calyceal fat noted through the injury. In the SMP group, only one patient had minor mucosal calyceal injury. Intraoperative calyceal injury was lower in the SMP group, although it was statistically insignificant (*p* = 0.42). Three patients in the SMP group and one patient in the standard group underwent a totally tubeless PCNL (no double-J stent or nephrostomy tube). None of the patients from both groups in the present study had a nephrostomy tube.

**Table 2:** Comparative statistics of standard PCNL and SMP

		Standard PCNL	SMP	*P* value
Efficacy				
Stone-free rate ( %)		94%	98%	0.14
Intraoperative complications—calyceal injury				
Minor mucosal tear		5	1	0.42
Major mucosal laceration		1	0	
Fat seen (pericalyceal)		1	0	
Procedure time				
Mean operative time (in min)		35.6 ± 6.8	51.62 ± 10.17	< 0.00001
Postoperative complications ( modified Clavien-Dindo classification)				
Grade I	Transient fever	5 (10%)	1 (2%)	0.096
Grade II	Haematuria (not requiring transfusion)	2 (4%)	0	0.49
Grade III	Pneumothorax	1 (2%)	0	1.0
Grade IV/V	N/A	0	0	
Operative blood loss				
Mean difference in pre- and postoperative Hb levels (g %)		1.2 ± 0.81	0.8 ± 0.7	0.021
Visual analogue score for postoperative pain (out of 10)				
Immediate postoperative period		5.7 ± 0.9	5.4 ± 0.7	0.03
6 h		4.3 ± 1.0	4.1 ± 0.7	
12 h		2.7 ± 0.8	2.5 ± 0.6	
24 h		1.4 ± 0.6	1.4 ± 0.6	
Hospital stay				
Mean duration of hospital stay (h)		39.84	28.38	0.0001

Transient fever in the postoperative period requiring antipyretics was noted in five patients in the standard group and in one patient in the SMP group (Clavien I). Persistent haematuria occurred in two patients in the standard group lasting for 8 h in each case, without the need for blood transfusion or intervention. One patient in the standard group developed pneumothorax, which was managed with an ICD insertion (Clavien III).

The difference in haemoglobin drop between the two groups was not statistically significant (*p* = 0.21). Mean (± SD) pain VAS scores recorded in the immediate postoperative period (5.7 ± 0.7 vs 5.9 ± 0.9), 6 h postop (4.3 ± 1.0 vs 4.1 ± 0.7), 12 h postop (2.7 ± 0.8 vs 2.5 ± 0.6) and 24 h postop (1.4 ± 0.6 vs 1.4 ± 0.6) showed a significantly lower VAS score in the SMP group (*p* = 0.03) during the immediate postoperative period and similar VAS scores at 24 h postop. The mean analgesic requirement was significantly lower in SMP group (89 mg tramadol vs 124.3 mg tramadol, *p* = 0.0001). The mean (SD) hospital stay was noted to be 39.84 (3.7) h in the standard group and 28.38 (3.6) h in the SMP group, respectively, which was significantly lesser in the SMP group (*p* = 0.0001). Stone-free rates in the standard group was 94%, while in the SMP group it was 98% (*P* = 0.14).

## Discussion

PCNL remains the preferred endourological modality for the management of renal stones larger than 2 cm and has demonstrated superior efficacy, particularly for addressing lower pole stones larger than 1 cm [4, 5]. However, it is the most invasive option and carries a risk of serious complications, which limits its utilization [6]. Guidelines state that miniaturized PCNL is associated with a lower complication rate when compared to standard PCNL without compromising the SFR [4, 7]. The tract sizes for miniaturized procedures are generally accepted to range from 4.8 F to 22 F. This includes mini PCNL (14–22F), ultramini PCNL (11–13F) and micro-PCNL (4.85–10F) [7–9]. While miniaturized PCNL has been recommended to reduce morbidity, there is no definitive or standardized tract size for various stone sizes, and ultimately, the choice depends on the surgeon's discretion. Studies suggest that miniaturized PNL appears to be more effective for smaller renal stones rather than larger ones, especially those exceeding 20 mm in size [10], when compared to standard PCNL. This holds true particularly for ultramini-PCNL [9] and micro-PCNL[8].

SMP demonstrates superior efficacy for smaller renal stones (2–3 cm) [11], while its effectiveness diminishes for larger stones (> 40 mm), leading to decreased SFR, prolonged operative times, and a tendency towards metabolic acidosis [11, 12]. The utilization of enhanced SMP (18 Fr) for treating larger renal calculi aiming to achieve a higher stone clearance rate while reducing operative time compromises the advantage of a small tract size, resulting in a perioperative risk profile almost comparable to standard PCNL [13]. This is corroborated by the findings of an experimental study examining renal parenchymal injury following PCNL tract dilatations in cadaveric kidneys, where single-step dilations up to sizes 8.5/9.5 Fr to 16.5/17.5 Fr tracts resulted in only 1-mm capsular retraction and fissures, whereas larger tracts up to 21/22 Fr showed renal rupture and capsular retraction, and the dilatation tract exceeded the device area. Therefore, larger tracts during PCNL may increase the vulnerability of kidneys to haemorrhagic complications [14].

In this study, the mean stone size and volume were similar between the SMP group and the standard PCNL group, thereby enhancing the comparability between the two groups. Stone size, stone type, tract size and surgeons’ experience are the major factors affecting operative time in PCNL [15, 16]. In our study, the mean operation time was significantly higher in the SMP group when compared to the standard group, which could be attributed to the increased time needed for dusting the stone and extracting residual fragments through a smaller size tract. However, despite the prolonged operative times, there was no significant increase in postoperative fever, sepsis and haemorrhagic complications in the SMP group as compared to the standard group in our study.

Due to the use of a smaller tract, reduced bleeding and active suction– evacuation, providing enhanced stone fragment retrieval, the omission of placing a double-J stent was more favourable in the SMP group, leading to a higher proportion of patients undergoing a totally tubeless procedure. None of the patients in the study had a nephrostomy tube placed. Prior studies on SMP have reported a high rate of 'tubeless' and 'stent-free' PCNL in the SMP group [17]. The advantages of tubeless and stent-free procedures include decreased patient discomfort, fewer lower urinary tract symptoms and sexual dysfunction, reduced postoperative analgesic needs, shorter hospital stay and avoidance of a second procedure for stent removal [18–20].

Collecting system injury in PCNL is reported in up to 5.2% of cases, whereas urinoma formation occurs in only 0.2% of cases [6]. Intraoperative calyceal injuries were lower in the SMP group reflecting the reduced risk of calyceal injury with SMP, possibly due to smaller sheath size and single-step dilatation minimizing tissue violation and manipulation during access, especially in patients with narrow infundibula.

Studies report post-PCNL transfusion rates ranging from 0 to 20%, with an overall rate of 7% [6]. Factors associated with increased risk of haemorrhagic complications include multiple punctures, dilation with a large access sheath [14], the presence of large stones and prolonged operative time [21]. In this study, no statistically significant drop in haemoglobin was observed postoperatively between the two groups. This may be attributed to the fact that standard PCNL procedures were conducted by a single experienced surgeon, which probably lowered the anticipated drop in postoperative haemoglobin levels. In our study, two patients in the standard PCNL group had persistent haematuria for 8 h, which resolved without blood transfusion or additional intervention. No patients in the SMP group experienced postoperative persistent haematuria, highlighting SMP's advantage in reducing haemorrhagic complications.

Fever following PCNL is a common occurrence with an incidence of 10.8% [6], which could be attributed to inflammation, possibly induced by larger tracts or stones harbouring infection. While smaller tract sizes are associated with an elevated risk of increased intrarenal pressures, predisposing patients to fever and sepsis [22], the use of continuous suction–evacuation in SMP helps maintain low intrarenal pressure [13], and continuous evacuation of stone fragments can reduce their stagnation within the pelvicalyceal system, thereby decreasing the duration of bacterial exposure. This possibly explains the reduced occurrence of postoperative febrile complications in the SMP group in our study.

Thoracic complications of PCNL are uncommon (less than 2%), more frequently associated with supracostal punctures [23–25]. In the standard group, one patient with a supracostal access developed pneumothorax and was managed with intercostal drain insertion. No patients in the SMP group, even with supracostal access, encountered thoracic complications, possibly due to the smaller tract size causing minimal pleural violation, resulting in smaller pleural injury with a higher likelihood of spontaneous closure following access tract removal.

In our study, the postoperative static and dynamic pain score was significantly lower in the SMP group during the immediate postoperative period, potentially attributed to the smaller tract size. The reduced incidence of postoperative complications and improved pain profile in the SMP group are reflected by a significantly shorter hospital stay compared to the standard group, similar to the findings reported by previous studies [26].

When discussing the efficacy of PCNL, it refers to the ability to achieve the highest stone-free rates. Comparing stone-free rates is challenging due to varied definitions for stone-free status, criteria for accepting fragments and differences in imaging modalities used, ranging from ultrasound and X-ray KUB to CT [27]. RCTs comparing SMP to standard PCNL for stones < 2 cm [26] or > 2 cm [28] and SMP to mni PCNL for stones > 2 cm [13] have demonstrated high stone clearance rates of SMP. In our study, standard group achieved a stone clearance rate of 94%, while the SMP group demonstrated a notably higher rate of 98%, indicating efficient stone clearance in the SMP approach for stones up to 3 cm in size. SMP distinguishes itself in fragment removal compared to UMP and micro PCNL. While UMP relies on a whirlpool effect or leaf fragments for spontaneous passage, as does micro PCNL, SMP actively removes stone fragments using continuous negative suction pressure and simultaneous use of a 3 Fr grasper, particularly beneficial for hard stones, resulting in superior stone clearance rates for stones > 2 cm [8, 9, 11, 17].

The strengths of our study include randomized controlled design with comparable mean stone sizes in both arms, addressing common renal stone sizes in the current clinical scenario. However, limitations include reliance on X-ray KUB and renal USG for postoperative fragment assessment instead of CT scan, potentially limiting accuracy.

We did not utilize scoring systems for stone complexity assessment in our study. Absence of distinct documentation for fragmentation and stone retrieval times is another limitation of the study. Also, as the procedure is done by a single surgeon, reproducibility of the outcomes can be questionable. Future studies should incorporate nomograms, assess quality of life and conduct cost–benefit analysis [29, 30].

## Conclusion

Super-mini PCNL is equally effective as standard PCNL in treating renal calculi up to 3 cm, with significantly reduced postoperative pain and duration of hospital stay with lower risk of high-grade complications.

## Data Availability

Not applicable.
